# 2026 Acknowledgment of Microbiology Spectrum Reviewing Editors

**DOI:** 10.1128/spectrum.00668-26

**Published:** 2026-05-05

**Authors:** Christina A. Cuomo

**Affiliations:** 1 Brown University 6752, Providence, Rhode Island, USA

## EDITORIAL

The Reviewing Editor program at *Microbiology Spectrum* is open to early-career microbiologists from across the globe. The Reviewing Editor role is distinct from the Editor role in that, while Editors choose peer reviewers and make decisions on manuscripts, Reviewing Editors serve as expert reviewers for one to two papers per month (a maximum of 24 per year) and receive feedback on their reviews. Reviewing Editors advance to the Editor role based on their performance and subject area expertise needs for Editors at the journal.

Currently, we have 129 Reviewing Editors from 27 countries with expertise across microbiology. In the past year, our Reviewing Editors provided nearly 450 reviews for the journal—extraordinary service for which we are very grateful. Furthermore, 35% of all manuscripts were evaluated by at least one Reviewing Editor.

This past year, we continued to promote Reviewing Editors to the Editor role. These individuals were selected based on their subject area expertise, number of reviews completed, and the quality of the reviews. We also welcomed a new cohort of Reviewing Editors to the Board, including applicants through our Open Board process.

We thank all our Reviewing Editors, past and present, for being part of this program. Please join us in congratulating the following people who served in the Reviewing Editor role in 2025 (Reviewing Editors who were promoted to the Editor role are bolded):

Rachy Abraham

Nurul Ahmad

Holly Arnold

Arianna Basile

**Arden Baylink**

**Michael Becker**

Saina Beitari

**Francesca Benedetti**

Banhi Biswas

Sébastien Bontemps-Gallo

Gillian Bruni

Weiwei Cai

Cecilia Carvalhaes

Emily Chase

Alexandra Chiaverini

Francesco Comandatore

Jôice Corrêa

Claudio Da Silva

Maria Célia Da Silva Lanna

Bismarck Dinko

Erin Eggleston

Dina El-Ghonemy

Paulo Fernandes Junior

Gianmarco Ferrara

Carlos Figueroa Castro

Javier Gandasegui

Ketaki Ganti

Peng Gao

Muluwork Getahun

Heather Glasgow

Shan Goh

Payal Gupta

Hajar Hazime

Shannon Hinsa-Leasure

Yaoqin Hong

Yujie Hu

Wei Huang

Yosef Huberman

Kelly Ishida

Shilpi Jain

Ghulam Jeelani

Hyung Jong Jin

Teny John

Jan Naseer Kaur

Jaspreet Kaur

Johanna Kenyon

Rohitashw Kumar

Krishna Kurthkoti

Dexi Li

Hao Li

Wentao Li

George Liechti

Xiaoyan Lin

Qin Liu

**Veranja Liyanapathirana**

Ti Lu

**Mor Lurie-Weinberger**

Michael Macey

Joseph Madison

Shankar Manoharan

Holly Martin

Bruno Martorelli Di Genova

Bridget McGivern

Sarita Mohapatra

Shahzad Munir

Caitlin Murphy

Azdayanti Muslim

Kang Ning

Myat Nyunt

Ezgi Ozcan

Ayon Pal

Ajit Passari

Bethany Patenall

Helena Pětrošová

Chin-Soon Phan

Cécile Philippe

Camilla Pires

Parmanand Prabhakar

Vibhu Prasad

**Elias Rahal**

Carlos Ramírez-Flores

Guanhua Rao

Vishwanatha Reddy

**Christopher Rice**

Sanghamitra Saha

Jennifer Salerno

Dhritiman Samanta

André Santos

Yoshiaki Sato

Allison Scherer

J. Dylan Shropshire

**Jaime Soria**

Vaibhav Srivastava

Karthik Subramanian

Hao Tan

Li Tao

Maria Tomas

**Xinzhao Tong**

Deeksha Tripathi

Gareth Trubl

Pablo Tsukayama

Filipa Vale

Juan Vazquez-Ucha

Benjamin Von Bredow

Crista Wadsworth

Caray Walker

Zhenzhou Wan

Fang Wang

Wei Wang

Xiaogang Wang

Yan Wang

Yiming Wang

Yahan Wei

Zhu Xiaoyu

Yifei Xu

Yunjian Xu

Chao Yang

Feng Yang

Jian Yang

Jinki Yeom

Yongchao Yin

Sunghyun Yoon

Changqing Yu

Manda Yu

Changyi Zhang

Zhemin Zhang

Zhen Zhang

Yuchao Zhao

Chunyi Zhou

